# Care Gaps in Sodium-Glucose Cotransporter-2 Inhibitor and Renin Angiotensin System Inhibitor Prescriptions for Patients with Diabetic Kidney Disease

**DOI:** 10.1007/s11606-022-07863-0

**Published:** 2022-11-09

**Authors:** Sharon Rikin, Stephanie Deccy, Chenshu Zhang, Jill Crandall, Yuting Deng, Ladan Golestaneh

**Affiliations:** 1 Division of General Internal Medicine, Montefiore Medical Center and Albert Einstein College of Medicine, 3300 Kossuth Avenue, Bronx, NY 10467 USA; 2 Montefiore Medical Center and Albert Einstein College of Medicine, 3300 Kossuth Avenue, Bronx, NY 10467 USA

**Keywords:** chronic kidney disease, evidence-based medicine, diabetes mellitus, sodium-glucose cotransporter-2 inhibitor, renin and angiotensin system inhibitor

## Abstract

**Background:**

Renin and angiotensin system inhibitors (RAASi) and sodium-glucose cotransporter-2 inhibitors (SGLT2i) are recommended for patients with diabetic kidney disease (DKD) to reduce the progression to end-stage kidney disease; however, they are under-prescribed.

**Objective:**

To evaluate the frequency of care gaps in RAASi and SGLT2i prescription by patient demographic, health system, and clinical factors in patients with DKD.

**Design:**

Retrospective cohort study.

**Participants:**

Adult primary care patients with DKD at an integrated health system in Bronx, NY, with 23 primary care sites in 2021.

**Main Measures:**

The odds of having a care gap for (1) SGLT2i or (2) RAASi prescription. Multivariate logistic regression models were performed for each outcome measure to evaluate associations with patient demographic, health system, and clinical factors.

**Key Results:**

Of 7199 patients with DKD, 80.3% had a care gap in SGLT2i prescription and 42.0% had a care gap in RAASi prescription. For SGLT2i, patients with A1C at goal (aOR 2.32, 95% CI 1.96–2.73), Black non-Hispanic race/ethnicity (aOR 1.46, 95% CI 1.15–1.87), and Hispanic race/ethnicity (aOR 1.46, 95% CI 1.11–1.92) were more likely to experience a care gap. For RAASi, patients with blood pressure at goal (aOR 1.34, 95% CI 1.21–1.49) were more likely to experience a care gap.

**Conclusions:**

The care gaps for SGLT2i and RAASi for patients with DKD with well-controlled diabetes and blood pressure suggest failure to recognize DKD as an independent indication for these medications. Racial/ethnic disparities for SGLT2i, but not for RAASi, suggest systemic racism exacerbates care gaps for novel medications. These factors can be targets for interventions to improve patient care.

## Introduction

In the USA, over 12 million individuals have diabetic kidney disease (DKD) and 38% of end-stage kidney disease (ESKD) is caused by diabetes.^1–3^ There is longstanding evidence supporting use of renin and angiotensin system inhibitors (RAASi) to slow the progression of DKD to ESKD.^4–6^ More recently, sodium-glucose cotransporter-2 inhibitors (SGLT2i) have demonstrated reduction of ESKD or death due to CKD in patients with diabetes.^7–11^ However, studies for patients with DKD reveal care gaps for both RAASi and SGLT2i prescriptions.^12–16^

Clinical practice guidelines recommend RAASi and SGLT2i for patients with DKD.^17–19^ The Kidney Disease Improving Global Outcomes (KDIGO) and American Diabetes Association guidelines recommend RAASi for diabetic patients with albuminuria, independent of blood pressure control, and SGLT2i for diabetic patients with CKD, regardless of glycemic control.^18,19^ RAASi prescription increased in the early 2000s, but plateaued in the last decade; estimates of RAASi prescription are 32–58% in people with diabetes and CKD.^2,16^ SGLT2i prescriptions have increased over time; however, estimates of SGLT2i use remain <10% in people with diabetes and CKD between 2015 and 2019.^2,13,14^ Furthermore, there are disparities based on older age, non-white race, female gender, and low income.^13,14,16^

Previous studies examining care gaps have primarily focused on patient demographics. Prescribing may also be influenced by clinical factors such as glycemic control, blood pressure control, medication burden, or hyperkalemia^20^ and health system factors such as patient’s engagement in primary care.^21–23^ Insurance formulary restrictions and requirement of prior authorization may disincentivize prescribing, particularly for SGLT2i which are not available in generic formulations. Individual provider’s practice type or whether they are a resident physician may impact behaviors such as early adoption of novel medications or familiarity with evidence-based guidelines.^22,23^

The objective of this study was to evaluate the frequency of care gaps in RAASi and SGLT2i prescription by patient demographic, health system, and clinical factors in patients with diabetic kidney disease engaged in primary care. Identification of factors that obstruct implementation is essential to improving evidence-based care for patients to prevent progression of DKD to ESKD.

## Methods

### Study Design, Setting, and Participants

This is a retrospective cohort study of patients with DKD at Montefiore Medical Center (MMC), an integrated health system with 23 primary care locations in Bronx, NY, serving mostly publicly insured patients. MMC had quality improvement infrastructure to support and incentivize evidence-based care for diabetes; however, there was no focus on DKD such as clinical decision support for nephropathy screening or prescribing DKD-related medications. Support for insurance prior authorization and collaboration with clinical pharmacists were available. This study was part of a new DKD quality initiative which began with evaluating baseline implementation of evidence-based care.

Using the Epic electronic health record (EHR), we developed a registry of patients (age, ≥ 18 years) with DKD meeting the following criteria: (1) appointment with MMC primary care provider (PCP) between January 12, 2021, and January 12, 2022, (2) diagnosis of type 2 diabetes, (3) proteinuria on laboratory test from July 12, 2021, to January 12, 2022 (microalbumin/creatinine ratio, urine ≥ 30 mg/g; total protein/creatinine ratio, urine ≥ 50 mg/g; total protein, timed urine ≥ 50 mg/24 h; protein urine random ≥ 1+ or > 30 mg/g; or protein, point-of-care urine ≥ 30 mg/g), (4) no diagnosis of ESKD. All patients in the registry met criteria for RAASi and SGLT2i prescription according to evidence-based guidelines. This study was approved by the Albert Einstein College of Medicine and MMC Institutional Review Board.

### Measures

The two outcome measures were the odds of having a care gap for medication in the pharmaceutical classes (1) SGLT2i and (2) RAASi. A care gap was defined as not having an active prescription in the EHR on the date of the data extraction.

Covariates included factors known to influence prescribing of SGLT2i and RAASi as well as clinical considerations for diabetes and hypertension management. Patient demographic factors included age, sex, and self-identified race and ethnicity (White non-Hispanic, Black non-Hispanic, Asian non-Hispanic, Hispanic, or other, which included patients who specified “Other” as their race, as well as patients without race/ethnicity information available or patients who declined to provide race/ethnicity information). Health system factors included insurance type (commercial, public or self-pay; self-pay was combined with public because patients at Federally Qualified Health Centers may receive prescription assistance), name of patient’s PCP, and whether the PCP was a resident physician or based at a teaching site. Clinical factors included mean eGFR from June 12, 2021, to January 12, 2022 (GFR was estimated using the modified CKD-EPI equation which does not adjust for race and categorized based on KDIGO CKD guidelines: stage 1, ≥ 90 mL/min/1.73 m^2^; stage 2, 60 to 89; stage 3, 30 to 59; and stage 4, < 30), most recent hemoglobin A1C (≤ 7 or not at goal), most recent blood pressure (systolic blood pressure ≤ 130 or not at goal), most recent potassium (K <5.5 or not in normal range), hyperkalemia (K ≥ 5.5) ever recorded in the medical record, presence of severe proteinuria (microalbumin/creatinine ratio, urine > 300 mg/g; total protein/creatinine ratio, urine > 300 mg/g; total protein, timed urine > 300 mg/24 h; protein urine random >3+ or >00 mg/g; or protein, point-of-care >300 mg/g), and number of additional unique prescriptions related to diabetes (EHR-defined pharmaceutical classes including insulins, biguanides, GLP-1 agonists, DPP4 inhibitors, sulfonylureas, thiazolidinediones, and alpha glucosidase inhibitors) and separately hypertension (EHR-defined pharmaceutical classes including thiazide, loop and potassium-sparing diuretics, beta blockers, calcium channel blockers, combined alpha and beta blockers, alpha antagonists, hydralazine).

### Statistical Analysis

Descriptive statistics were used for patient demographic, clinical, and health system characteristics, as well as the prevalence of care gap for SGLT2i or RAASi prescription. To build a parsimonious predictive model, we strategically included covariates and tested their association with the outcomes of interest using bivariate analyses controlling for individual PCP as a random effect variable. A multivariate GEE logistic model controlling for individual PCP as a random effect variable was built for each outcome measure, retaining significant variables (*p* value ≤ 0.05 on bivariate testing), retaining nonsignificant variables that were known to be associated with prescriptions, and eliminating variables that were both nonsignificant and not known to be associated with prescriptions. Post hoc bivariate analyses were conducted for preferred language (English vs. other). Due to strong evidence for SGLT2i or RAASi prescription in patients with severe proteinuria, sensitivity analyses were conducted to investigate covariates associated with care gaps in this population. Post hoc pairwise analyses for GFR categories were conducted for each outcome measure to further elucidate the role of CKD in prescribing. Data were derived from a cohort of patients engaged in care during the 365 days prior to the data extraction and analyzed in January 2022.

## Results

There were 7199 patients with DKD; 5780 patients (80.3%) were not prescribed SGLT2i, and 3022 patients (42.0%) were not prescribed RAASi at the time of the data extraction. All patients had at least one PCP visit during the study period and 92.3% attended the most recent PCP visit in-person. Patient demographic, clinical, and health system characteristics are found in Table 1 and prevalence of care gaps by select characteristics in Table 2. In bivariate testing, the following factors were associated with SGLT2i prescription: age, sex, race/ethnicity, having a resident physician as PCP, insurance type, CKD stage, hemoglobin A1C, severity of proteinuria, number of other diabetes medications; this nonsignificant factor was retained for the multivariate model: receiving care at a teaching site. In bivariate testing, the following factors were associated with RAASi prescription: age, CKD stage, blood pressure, history of hyperkalemia, severity of proteinuria, number of other hypertension medications; these nonsignificant factors were retained for the multivariate model: sex, race/ethnicity, insurance type, having a resident physician as PCP, receiving care at a teaching site. In post hoc bivariate analyses, preferred language was not associated with either SGLT2i or RAASi prescription.

**Table 1:** Patient Demographic, Clinical, and Health System Characteristics

Variable	**Mean, SD**
Age	67.3, 13.1
	***N*, %**
Sex	
Female	4209, 58.5%
Race/Ethnicity	
White, non-Hispanic	410, 5.7%
Black, non-Hispanic	2702, 37.5%
Asian	254, 3.5%
Hispanic	2913, 40.5%
Other, unspecified	920, 12.8%
Insurance type	
Commercial	1548, 21.5%
Public	5651, 77.5%
Self-pay	74, 1.03%
PCP site type	
Non-teaching site	5090, 70.7%
Teaching site	2109, 29.3%
PCP training level	
Resident physician as PCP	809, 11.2%
Non-resident healthcare provider as PCP	6390, 88.8%
HbA1c	
At goal, ≤ 7	3395, 47.2%
Not at goal	3804, 52.8%
Blood pressure	
At goal ≤ 130/80	2355, 32.7%
Not at goal	4844, 67.3%
GFR categories	
Category 1, GFR ≥ 90	1461, 20.3%
Category 2, GFR 60–89	2996, 41.6%
Category 3, GFR 30–50	2250, 31.3%
Category 4, GFR < 30	492, 6.8%
Degree of proteinuria	
Moderate proteinuria	5488, 76.2%
Severe proteinuria	1711, 23.8%
Potassium (K)	
Normal, K < 5	6463, 89.8%
K abnormal or unknown	736, 10.2%
History of hyperkalemia (K > 5.5)	
Hyperkalemia ever	1939, 26.9%
Hyperkalemia never	5260, 73.1%
Count of other diabetes-related Rx *	
0	1251, 17.4%
1	2239, 31.1%
2	2104, 29.2%
3	1275, 17.7%
4	291, 4.0%
5+	39, 0.5%
Count of other hypertension-related Rx ^†^	
0	2591, 36.0%
1	2595, 36.0%
2	1479, 20.5%
3	417, 5.8%
4	97, 1.3%
5+	20, 0.3%

*Count of prescriptions in EHR-defined pharmaceutical classes: insulins, biguanides, GLP-1 agonists, DPP4 inhibitors, sulfonylureas, thiazolidinediones, and alpha glucosidase inhibitors; excludes SGLT2i prescription

†Count of prescriptions in EHR-defined pharmaceutical classes: thiazide, loop and potassium-sparing diuretics, beta blockers, calcium channel blockers, combined alpha and beta blockers, alpha antagonists, hydralazine; excludes RAASi prescription

Patients with missing data for (1) race/ethnicity were coded as “Other,” unspecified (*N*=301), (2) HbA1C were coded as not at goal (*N*=17), (3) blood pressure were coded as not at goal (*N*=3), and (4) potassium were classified as K ≥ 5 (*N*=63)

**Table 2:** Prevalence of Care Gaps for Select Patient Characteristics

Variable	Care gap for SGLT2i Rx	Care gap for RAASi Rx
	***N*, %**	***N*, %**
Sex		
Female	3510, 83.4%	1794, 42.6%
Male	2270, 75.9%	1228, 41.1%
Race/Ethnicity		
White, non-Hispanic	306, 74.6%	166, 40.5%
Black, non-Hispanic	2194, 81.2%	1171, 43.3%
Asian	189, 74.4%	100, 39.4%
Hispanic	2357, 80.9%	1233, 42.3%
Other, unspecified	734, 79.8%	352, 38.3%
Insurance type		
Commercial	4554, 81.7%	635, 41.0%
Public	1163, 75.1%	2360, 42.3%
Self-pay	63, 85.4%	27, 36.5%
HbA1c		
At goal, ≤ 7	2994, 88.2%	--
Not at goal	2768, 73.2%	--
Blood pressure		
At goal, ≤ 130/80	--	1900, 39.2%
Not at goal	--	1122, 47.6%
GFR categories		
Category 1, GFR ≥ 90	1175, 80.4%	683, 46.8%
Category 2, GFR 60–89	2502, 83.5%	1172, 39.1%
Category 3, GFR 30–50	1696, 75.4%	894, 39.7%
Category 4, GFR < 30	406, 82.5%	273, 55.5%
Degree of proteinuria		
Moderate proteinuria	4502, 82.1%	2350, 42.8%
Severe proteinuria	1277, 74.6%	672, 39.3%

### Care Gap in SGLT2i Prescription Multivariate Model

Demographic factors: Males were less likely to experience a care gap in SGLT2i compared to females (aOR 0.69, 95% confidence interval [CI] 0.61–0.78). Black non-Hispanic and Hispanic patients were more likely to experience a care gap compared to White non-Hispanic patients (aOR 1.46, CI 1.15–1.87, for Black non-Hispanic; and aOR 1.46, CI 1.11–1.92 for Hispanic). In addition, patients categorized as “Other” race were more likely to experience a care gap compared to White non-Hispanic patients (aOR 1.40, CI 1.07–1.84). Health system factors: Patients with commercial insurance were less likely to experience a care gap compared to publicly insured patients (aOR 0.78, CI 0.64–0.95). Clinical factors: Patients with A1C at goal were more likely to experience a care gap compared to patients with A1C not at goal (aOR 2.32, CI 1.96–2.73). Patients with moderate proteinuria were more likely to experience a care gap compared to patients with severe proteinuria (aOR 1.37, CI 1.15–1.63). Patients with GFR between 30 and 59 were less likely to experience a care gap compared to patients with GFR ≥ 90 (aOR 0.54, CI 0.41–0.72). In addition, for every additional prescription related to diabetes, patients were less likely to experience a care gap (aOR 0.83, CI 0.77–0.90). The aORs for all variables included in the model are provided in Table 3.

**Table 3:** Multivariate GEE Logistics Model of Factors Associated with Care Gaps in SGLT2i Prescription*

Variable	AOR (95% CI)	*p* value
Age	1.01 (1, 1.02)	< 0.001
Sex		
Female	Ref	
Male	0.69 (0.61, 0.78)	< 0.0001
Race/ethnicity		
White, non-Hispanic	Ref	
Asian	1.02 (0.72, 1.43)	0.93
Black, non-Hispanic	1.46 (1.15, 1.87)	< 0.01
Hispanic	1.46 (1.11, 1.92)	< 0.01
Other race/ethnicity	1.40 (1.07, 1.84)	0.01
Insurance type		
Public or self-pay	Ref	
Commercial	0.78 (0.64, 0.95)	0.02
PCP site and type		
Non-teaching site	Ref	
Teaching site, resident as PCP	0.85 (0.65, 1.10)	0.21
Teaching site, non-resident as PCP	1.10 (0.82, 1.47)	0.52
HbA1C		
At goal, ≤ 7	Ref	
Not at goal	2.32 (1.96, 2.73)	< 0.0001
Proteinuria		
Severe proteinuria	Ref	
Moderate proteinuria	1.37 (1.15, 1.63)	< 0.001
GFR categories		
Category 1, GFR ≥ 90	Ref	
Category 2, GFR 60–89	0.94 (0.73, 1.19)	0.59
Category 3, GFR 30–50	0.54 (0.41, 0.72)	< 0.0001
Category 4, GFR < 30	0.82 (0.55, 1.20)	0.3
Count of other diabetes-related Rx^†^	0.83 (0.77, 0.90)	< 0.0001
HbA1C		
At goal, ≤ 7	Ref	
Not at goal	2.32 (1.96, 2.73)	< 0.0001
Proteinuria		
Severe proteinuria	Ref	
Moderate proteinuria	1.37 (1.15, 1.63)	< 0.001

*Model adjusted for individual PCP as a random effect variable

†Continuous variable, count of prescriptions in EHR-defined pharmaceutical classes: thiazide, loop and potassium-sparing diuretics, beta blockers, calcium channel blockers, combined alpha and beta blockers, alpha antagonists, hydralazine

In a sensitivity analysis of patients with severe proteinuria, more pronounced care gaps were observed in those with A1C at goal compared to not at goal (aOR 2.20, CI 1.72–2.80). Post hoc pairwise analysis by GFR category (Table 4) demonstrated that compared to patients with GFRs between 30 and 59, patients with normal GFRs ≥ 90 were more likely to experience a care gap in SGLT2i prescription (aOR 1.84, CI 1.39–2.45). In addition, patients with minimally decreased GFRs between 60 and 89 were more likely to experience a care gap compared to patients with GFR between 30 and 59 (aOR 1.73, CI 1.45–2.06).

**Table 4:** Care Gap in SGLT2i Prescription GFR Post Hoc Pairwise Analysis

	Ref. category 2	*p* value	Ref. category 3	*p* value	Ref. category 4	*p* value
	aOR (95% CI)		aOR (95% CI)		aOR (95% CI)	
Category 1, GFR ≥ 90	1.07 (0.84, 1.36)	0.59	1.84 (1.39, 2.45)	< 0.0001	1.23 (0.83, 1.81)	0.30
Category 2, GFR 60–89	-	-	1.73 (1.45, 2.06)	< 0.0001	1.15 (0.86, 1.53)	0.34
Category 3, GFR 30–59	-	-	-	-	0.67 (0.51, 0.87)	< 0.01
Category 4, GFR < 30	-	-	-	-	-	-

### Care Gap in RAASi Prescription Multivariate Model

Demographic factors: There was no statistical difference observed between racial/ethnic groups in the likelihood of experiencing a care gap in RAASi prescription. Health system factors: Patients with commercial insurance were less likely to experience a care gap compared to publicly insured patients (aOR 0.85, CI 0.75–0.96). Clinical factors: Patients with blood pressure at goal were more likely to experience a care gap compared to patients with blood pressure not at goal (aOR 1.34, CI 1.21–1.49). Patients with moderate proteinuria were more likely to experience a care gap compared to patients with severe proteinuria (aOR 1.28, CI 1.14–1.44). Patients with GFR between 60 and 89 were less likely to experience a care gap compared to patients with GFR ≥ 90 (aOR 0.54, CI 0.41–0.72). Patients who had a history of hyperkalemia were more likely to experience a care gap compared to patients who had never experienced hyperkalemia (aOR 1.51, CI 1.35–1.69). In addition, for every additional prescription related to hypertension, patients were less likely to experience a care gap (aOR 0.86, CI 0.81–0.91). The aORs for all variables included in the model are provided in Table 5.

**Table 5:** Multivariate GEE Logistics Model of Factors Associated with Care Gaps in RAASi Prescription*

Variable	AOR (95% CI)	*p* value
**Age**	0.99 (0.98, 0.99)	< 0.0001
**Sex**		
Female	Ref	
Male	0.91 (0.82, 1.03)	0.13
**Race/ethnicity**		
White, non-Hispanic	Ref	
Asian	0.83 (0.59, 1.15)	0.25
Black, non-Hispanic	1.15 (0.92, 1.44)	0.23
Hispanic	1.03 (0.82, 1.28)	0.92
Other race/ethnicity	0.90 (0.69, 1.17)	0.42
**Insurance type**		
Public or self-pay	Ref	
Commercial	0.85 (0.75, 0.96)	< 0.01
**PCP site and type**		
Non-teaching site	Ref	
Teaching site, resident as PCP	0.81 (0.68, 0.97)	0.02
Teaching site, non-resident as PCP	0.90 (0.77, 1.05)	0.17
**Insurance type**		
Public or self-pay	Ref	
Commercial	0.85 (0.75, 0.96)	< 0.01
**Blood pressure**		
At goal ≤ 130/80	Ref	
Not at goal	1.34 (1.21, 1.49)	< 0.0001
**GFR categories**		
Category 1, GFR ≥ 90	Ref	
Category 2, GFR 60–89	0.84 (0.71, 0.99)	0.04
Category 3, GFR 30–50	0.94 (0.78, 1.13)	0.50
Category 4, GFR < 30	2.01 (1.54, 2.60)	< 0.0001
**Proteinuria**		
Severe proteinuria	Ref	
Moderate proteinuria	1.28 (1.14, 1.44)	< 0.0001
**Potassium**		
Normal, K < 5	Ref	
K abnormal or unknown	1.20 (0.99, 1.44)	0.06
**History of hyperkalemia**		
Never hyperkalemia	Ref	
Ever hyperkalemia	1.51 (1.35, 1.69)	< 0.0001
**Count of other hypertension-related Rx**^**†**^	0.86 (0.81, 0.91)	< 0.0001

*Model adjusted for individual PCP as a random effect variable

**†**Continuous variable, count of prescriptions in EHR-defined pharmaceutical classes: thiazide, loop and potassium-sparing diuretics, beta blockers, calcium channel blockers, combined alpha and beta blockers, alpha antagonists, hydralazine

In a sensitivity analysis of patients with severe proteinuria, similar trends were observed with respect to hyperkalemia (aOR 1.69, CI 1.36–2.10). However, blood pressure control was no longer associated with a care gap (aOR 1.19, CI 0.97–1.45). Post hoc pairwise analysis by GFR category (Table 6) demonstrated that compared to patients with minimally decreased GFRs between 60 and 89, patients with normal GFR ≥ 90 had a higher chance of experiencing a care gap (aOR 1.19, CI 1.01–1.40).

**Table 6:** Care Gap in RAASi Prescription GFR Post Hoc Pairwise Analysis

	Ref. category 2	*p* value	Ref. category 3	*p* value	Ref. category 4	*p* value
	aOR (95% CI)		aOR (95% CI)		aOR (95% CI)	
Category 1, GFR ≥ 90	1.19 (1.01, 1.40)	0.04	1.06 (0.88, 1.28)	0.50	0.50 (0.38, 0.65)	< 0.0001
Category 2, GFR 60–89	-	-	0.89 (0.79, 1.01)	0.07	0.42 (0.33, 0.52)	< 0.0001
Category 3, GFR 30–59	-	-	-	-	0.47 (0.39, 0.57)	< 0.0001
Category 4, GFR < 30	-	-	-	-	-	-

## Discussion

We found care gaps for RAASi and SGLT2i prescriptions for primary care patients with DKD. Despite clinical evidence of DKD, primary care patients with well-controlled diabetes, well-controlled hypertension, moderate proteinuria, and minimally decreased GFR were less likely to be prescribed SGLT2i and RAASi than those with uncontrolled diabetes or hypertension or more advanced CKD. This suggests there may be a lack of identification of early DKD or a lack of recognition of DKD as an indication for these medications independent from glucose or blood pressure management. In this predominantly Black and Hispanic community, we also found racial and ethnic disparities in prescriptions for SGLT2i, but not for RAASi, suggesting that systemic racism may exacerbate barriers to implementing novel evidence-based medications for patients of color.^24^ This study is the first to concurrently evaluate the prescription rates of RAASi and SGLT2i in a real-world cohort of primary care patients with DKD. Care gaps in our health system were lower than national estimates of patients with diabetes and CKD.^2,13,14,16^ For SGLT2i, differences in our cohort may be related to increased knowledge of the benefits of SGLT2i, increased insurance coverage over time, or differences in the patients sampled. Most previous studies were based on insurance claims databases while our study examined patients engaged in primary care.^2,13,14,16^

Inadequate initiation of evidence-based treatments is common in diabetes care and can be attributed to clinical inertia: a combination of provider, system-level, and patient barriers to guideline adherence.^25–29^ We found that some system-level factors impacted care gaps, such as insurance type, while others did not, such as having a resident physician as a PCP or receiving care at a teaching site. We found that males were less likely to experience a care gap for SGLT2i, possibly due to misperceptions regarding increased risk of genitourinary infections among women.^30,31^ Because of the complexity of primary care, there are three potential provider barriers to implementing evidence-based care.^32^ The first is failure to recognize DKD. In a multicenter study, for patients with diabetes, PCPs were more successful in detecting stage 3–5 CKD than those with stage 1–2.^33^ The second is lack of awareness that RAASi and SGLT2i are indicated for DKD independent of blood pressure or diabetes control.^17–19^ This can be explained by the evolution of guidelines over time; SGLT2i were initially recommended for glucose control, and only beginning in 2020 did the American Diabetes Association guidelines unequivocally recommend SGLT2i for DKD regardless of glucose control.^19,34,35^ The third is starting medications for long-term disease modification may not be a priority for PCPs or patients due to concerns of complex medication management, polypharmacy, or side effects. Unfortunately, in many cases there is a missed opportunity to identify DKD early enough to reduce progression and complications. Substantial gains against suffering, and towards cost savings, can be made if DKD is recognized early and treated aggressively. In an investigation of risk factors associated with CKD progression among Medicare Advantage Enrollees, it was found that kidney function declined more slowly in patients with clinical recognition of CKD, defined as the presence of ICD9/ICD10 diagnosis codes for CKD.^36^ Health system interventions such as patient registries or clinical decision support which prioritize early identification of DKD may help close care gaps.

Similar to our study, racial and ethnic disparities were found for the prescription of SGLT2i in a national study of commercially insured patients with diabetes^14^ and a study of patients with heart failure at our health system.^37^ Structural racism may uniquely impact the prescribing of novel therapies, explaining this finding for SGLT2i, but not for RAASi. For example, racial and ethnic disparities in continuous glucose monitoring and insulin pumps are well documented for patients with type 1 diabetes.^38–41^ Racial, ethnic, and socioeconomic inequalities have also been seen in the initiation of novel direct oral anticoagulant therapy for atrial fibrillation and venous thromboembolism.^42,43^ While more research is needed to elucidate the causes of disparities in our study, they may be explained by systemic racial inequities causing economic barriers such as affordability of novel brand name medication and cost of specialty care, or logistic barriers such as navigating insurance prior authorization.^24,44^ Patients of color may be more likely to worry about side effects, dependency, and cost of starting new medications or technologies which may be rooted in history of unfair healthcare treatment including experimentation with novel therapies.^45,46^ Providers may not offer novel medications due to an assumption that patients of color may be less likely to be accepting or may face more financial obstacles.^47,48^ Additionally, providers may not be skilled at employing shared decision-making with patients of color, particularly when there is patient-provider discordance.^47–50^ Black Americans comprise 13% of the US population, but more than 30% of the ESKD population progresses from CKD to ESKD at 3.5 times higher rate compared to white Americans.^1,2,51^ If this progression is even partially related to underutilization of evidence-based treatments, using a population health approach with attention to race and ethnicity has the potential to mitigate adverse DKD outcomes while improving health equity.

Our study has several strengths, including real-world data on both RAASi and SGLT2i prescriptions in a large population with racial and ethnic diversity and high prevalence of DKD. The integrated EHR allowed us to collect data on prescriptions, diagnosis codes, clinical data such as diabetes and blood pressure control, engagement in primary care, and prescriber information. Limitations include a single health system which may not be generalizable to other institutions. Because our outcome measures evaluated care gaps at one point in time, we are unable to determine the temporal relationship between factors and care gaps or which patients may have discontinued medications due to intolerance, adverse reactions (such as hyperkalemia or decrease in eGFR), or cost. Without detailed review of individual insurance pharmacy benefits, we do not know which patients faced barriers such as restrictive formularies, requirement for prior authorizations, or prohibitive cost-sharing. There may be misclassification in EHR race and ethnicity data as there is variation in how these data were elicited and entered. We plan to engage with PCPs and patients to determine the reason for identified care gaps and develop specific interventions to increase RAASi and SGLT2i prescriptions.

## Conclusions

This is the first study to contemporaneously evaluate factors associated with care gaps in prescription of SGLT2i and RAASi for patients with DKD. Despite primary care engagement, there were gaps in prescribing specifically for patients with well-controlled diabetes and hypertension, and early-stage CKD, suggesting that DKD and indications for these medications may be underrecognized by physicians and patients. Racial and ethnic disparities in prescriptions for SGLT2i must be addressed to improve health equity. We have identified targets for pragmatic interventions to increase prescription of evidence-based medications.
